# Clean Feet

**Published:** 1871-01

**Authors:** 


### CLEAN FEET.

The majority of people pay little attention to the cleanliness of the feet, and yet any square inch of the sole of the foot demands cleanliness, perfect cleanliness, more than any square foot of surface of the body, as far as health is concerned, because the "pores" are much larger there than anywhere else; so large indeed that they may be called "sluices" for carrying away the impurities of the system. Hence the bottom of the feet should be well washed and well rubbed every day.

